# The correlation between health status and quality of life in southern Brazil

**DOI:** 10.1590/S1516-31802008000500003

**Published:** 2008-09-04

**Authors:** Carlos Zubaran, Karina Persch, Desire Tarso, Ana Ioppi, Juan Mezzich

**Keywords:** Quality of life, Health Status, Questionnaires, Brazil, Health, Qualidade de vida, Nível de saúde, Questionários, Brasil, Saúde

## Abstract

**CONTEXT AND OBJECTIVE:**

The interconnections between quality of life and health status as assessed via questionnaires have not been thoroughly investigated. The objective of this study was to investigate a possible correlation between the constructs of general health status and quality of life as assessed by the Portuguese versions of two questionnaires recently adapted and tested in Brazil.

**DESIGN AND SETTING:**

This was a cross-sectional study in which two self-administered questionnaires were used. This investigation was conducted at healthcare services associated with the Universidade de Caxias do Sul, Brazil.

**METHODS:**

This study presents data from a sample of 120 volunteers who completed the Portuguese versions of the Personal Health Scale and the Multicultural Quality of Life Index questionnaires. Bivariate linear regression analysis and Pearson correlation coefficients were generated from the scores of the two questionnaires.

**RESULTS:**

A significant correlation between the concepts of quality of life and health status as evaluated by the Portuguese versions of both questionnaires was observed. Almost all of the health-related questions displayed strong correlations with the overall concept of quality of life. The magnitude of this correlation accounted for almost half of the observed variance.

**CONCLUSIONS:**

These findings indicate that, within this sample, health-related issues were key factors for the overall experience of wellbeing and quality of life. The similarities observed across the different groups indicate that the interrelation between health status and quality of life was homogenous, regardless of presence and/or type of ailments.

## RESUMO

**CONTEXTO E OBJETIVO:**

As interconexões entre qualidade de vida e estado de saúde não têm sido investigadas aprofundadamente. O objetivo deste estudo é investigar possível correlação entre construtos de estado geral de saúde e qualidade de vida mediante versões em português de dois questionários recentemente adaptados e testados no Brasil.

**TIPO DE ESTUDO E LOCAL:**

Estudo transversal com emprego de dois questionários auto-administráveis. Esta pesquisa foi conduzida em serviços de saúde associados à Universidade de Caxias do Sul, Brasil.

**MÉTODOS:**

Este estudo apresenta dados de uma amostra de 120 voluntários que completaram as versões em português da Escala de Saúde Pessoal e do índice Multicultural de Qualidade de Vida. Regressão linear bivariada e coeficientes de correlação de Pearson foram gerados a partir de escores de ambos questionários.

**RESULTADOS:**

Significativa correlação entre os conceitos de qualidade de vida e estado de saúde tais como avaliados pelas versões em português de ambos questionários foi observada. Quase todas as questões relacionadas à saúde da Escala de Saúde Pessoal apresentaram uma forte correlação com o conceito global de qualidade de vida. A magnitude desta significativa correlação foi responsável por quase metade da variação observada.

**CONCLUSÕES:**

Estes achados indicam que, nesta amostra, aspectos relacionados à saúde são essenciais para experiência global de bem-estar e qualidade de vida. Estas similaridades nos diferentes grupos indicam que a inter-relação entre estado de saúde e qualidade de vida é homogênea independentemente da presença e/ou tipo de doença.

## INTRODUCTION

During the latter part of the twentieth century, patients' subjective expressions became a salient topic in healthcare.^1^ The field of health status and quality of life measurement has been evolving as a formal discipline with structured theoretical foundations and specific methodology for more than 30 years.^2^ The concepts of health status, wellbeing and quality of life started to be conceived as researchable topics in the mid-1970s, when these constructs were often encompassed among psychosocial correlates of wellbeing and physiological factors.^3,4^ In the late 1980s, some authors adopted health screening procedures to examine the limitations imposed by the disease process on patients' wellbeing and social functioning.^5-8^ It was then understood that general health status is amenable to assessment and measurement and, thereafter, clinicians and researchers started to develop psychometric tools to evaluate a given patient´s impressions of his own health condition. The concept of personal health relates to the perception each individual has of his own health status.

Although the concept of health has been subject to cultural and historical adjustment, health can be defined as a state of complete physical, mental and social wellbeing according to the World Health Organization.^9-11^ Nonetheless, there is a current tendency to reexamine the conceptual boundaries of health in order to include additional elements such as sociocultural conditions, as well as factors contributing to mental and physical health, which ideally will transcend the rather circumscribed dichotomy of the health-disease process.^12^ It is currently acknowledged that efforts to investigate and evaluate quality of life, social functioning, health status and wellbeing are valid enterprises within clinical and research contexts.^13^

Quality of life has been defined as the individual's perception of his position in life in the context of cultural and value systems, in relation to his objectives, beliefs and expectations.^14^ For some, the concept of "health-related quality of life" is preferred over simply "quality of life" because the focus is on health, given that the health-related construct refers not only to physical, emotional and social wellbeing but more precisely to the "impact that health conditions and their symptoms have on an individual's quality of life, in the context of healthcare".^15^ The measurement of quality of life provides a benchmark against which the impact of disease and different treatments at personal level can be measured.^16^ An improvement in health status and quality of life is an important primary outcome in the determination of therapeutic benefit.^17^

Health status questionnaires, on the other hand, apart from providing parameters to monitor the impact of disease activity and the effect of a given therapeutic intervention, indicate the need for medical assistance and the degree of disability presented by a given patient. Health questionnaires are divided into two distinct domains of analysis, which are related to generic or disease-specific health measurements.^18^ General health instruments inquire about health in a broad sense, whereas disease-specific questionnaires assess narrower aspects of life relating to a specific problem, function or manifestation of an underlying disease process.^19^ Various generic health scales and questionnaires have been developed and adapted to an array of languages and cultures, including the Short Form 36-item Health Survey (SF-36), the Nottingham Health Profile (NHP), the Stanford Health Assessment Questionnaire (HAQ), the Modified Health Assessment Questionnaire (MHAQ), the EuroQol (EQ) and the Short Form 12-item Health Survey Questionnaire (SF-12).^20-25^

Clinicians and policymakers now agree on the importance of measuring both general and health-related quality of life.^26^ Among other applications, it has been demonstrated that general health questionnaires are useful tools for identifying episodes of emotional distress in general practice consultations.^27^ Due to the increasing numbers of multinational and multicultural research projects, there has been a proportional increase in the number of studies designed to test and adapt a series of health status instruments for use in different countries and languages.^28-30^ The majority of questionnaires were originally developed in English-speaking countries. However, the methodological details of translation and adaptation of questionnaires originally developed in English for future use in other countries have received surprisingly little attention.^31^ There is now a consensus that, when tested across different cultures, measurements must not only be well translated linguistically, but also be well adapted culturally in order to ensure the content validity of the instrument.^28,32-34^

The correlation between the concepts of quality of life and health status has been conceptualized as a continuum of complex outcomes in areas associated with patients' overall functioning, including physiological factors, symptomatology, overall functioning, general health perceptions and overall wellbeing.^35^ One of the few studies designed to investigate a possible correlation between health status and quality of life revealed no overlapping performance between a disease-specific quality of life questionnaire and a generic health assessment tool (SF-36) for assessing volunteers affected by allergic conditions.^36^ Nevertheless, the interconnections between the two constructs, along with the repercussions of possible construct communalities on structured assessments of quality of life and health status via psychometric tools still await further investigation.

## OBJECTIVE

The main objective of this study was to investigate a possible correlation between the constructs of health status and quality of life as assessed by the Portuguese versions of two questionnaires recently adapted and tested in Brazil. Details of the psychometric characteristics of the Portuguese versions of both tools can be obtained in their respective validation studies.^37,38^

## METHODS

### Subjects

This study investigated two samples of adult volunteers: one included 90 patients (30 from inpatient psychiatric units, 30 from outpatient facilities and 30 from general hospital units) and the other comprised 30 healthcare professionals from the same general hospital (total sample size = 120 research participants). The psychiatric units were located in a tertiary-care psychiatric hospital. Both the general hospital and the outpatient clinics were located on the main campus of Universidade de Caxias do Sul (UCS). All three healthcare services were also higher education training facilities.

All the patients who volunteered to participate in this investigation were attended via the Brazilian public health system (*Sistema Único de Saúde, SUS*). The healthcare professionals who voluntarily participated in this investigation were actively working nurses and nursing assistants who said they did not have any major health problems during a clinical interview prior to their enrollment in the study. All the professionals were employed under conventional working conditions and professional agreements. All the volunteers had Portuguese as their mother tongue.

### Informed consent

This study was endorsed by the institutional Research Ethics Committee of Universidade de Caxias do Sul. All the volunteers signed a consent form to declare their voluntary agreement with all procedures implicated in this project. Taking into account that a substantial fraction of the sample was illiterate or semi-illiterate, all patients completed the questionnaires under minimal guidance by trained examiners, who followed standardized instructional procedures.

### Procedures

Both the Personal Health Scale (PHS) and the Multicultural Quality of Life Index (MCQLI) were translated into Portuguese taking into account semantic, idiomatic, experiential, cultural and conceptual equivalence between the source and the target instruments.^34^ Two investigators proficient in both Portuguese and English developed the final Portuguese version of the PHS. Each investigator conducted the translation and adaptation of the questionnaires from one language to the other (translation and back-translation). The final adapted version of the instrument was established by a committee of specialists, by taking into consideration both the translation and the back-translation of each instrument. This committee consisted of professionals who were fully cognizant of the subject under investigation. Many of them were versed in both languages. The various drafts of both questionnaires, in each language, were progressively improved by using relevant information obtained from a series of applications of the questionnaires to various samples of patients and professionals.^14,39-41^

The Multicultural Quality of Life Index (MCQLI) is a visual analog scale composed of 10 dimensions, each one presenting unit values from 1 to 10. The dimensions include physical and emotional wellbeing, spiritual fulfillment, social functioning, community and services support and overall perception of life. Each dimension is to be rated by subjects according to their culture-informed understanding of that concept. The magnitude of the score parallels the intensity or quality of the construct, in that higher scores indicate an elevated perception of quality of life.

The Personal Health Scale (PHS) is a concise unidimensional instrument for comprehensive culture-informed and self-rated assessment of general health status. It contains 10 items that were obtained through critical review of the international literature, including factors such as sleep, mood, nervousness, fatigue and functional capacity. The Personal Health Scale is an ordinal scale that measures the frequency of ten dimensions and rates each one numerically from zero to three based on distinct levels of presence and/or frequency of a given phenomenon. The score magnitude of each domain parallels the frequency of each correlated dysfunction or symptom, such that the higher the overall score of the instrument is, the lower the personal health status is.

Both the MCQLI and the PHS were originally conceived and developed in the United States. Different versions of the questionnaires have already been validated in an array of languages.^39-41^ In the development of both psychometric tools, particular attention was paid to multicultural issues, so that specific conditions presented by immigrant groups are surveyed in the instrument.^33^ Copies of the Portuguese versions of the Personal Health Scale (PHS-Pt) and the Multicultural Quality of Life Index (MCQLI-Pt) can be obtained from the mailing author upon request.

Each patient completed both questionnaires administered: the PHS-Pt and the MCQLI-Pt. The overall score from each questionnaire for the entire sample, which was generated as the sum of the scores of each participant, was then correlated with the overall score from the other instrument. The overall score from one instrument was then correlated with each of the 10 questions in the other instrument. The resulting Pearson correlation coefficients are described below.

## RESULTS

### Sample characteristics

In the sample of patients, 45% of the volunteers were men. The patients' ages ranged from 20 to 70 years, with a mean of 47.7 years. In the sample of health professionals, 5% were men and the professionals' ages ranged from 20 to 59 years, with a mean of 37 years. All the health professionals were actively working during the investigational period.

### Pearson product-moment correlation coefficients

Correlation coefficients were computed between the overall scores (the sums of the scores of all ten questions from the two questionnaires) of PHS-Pt and MCQLI-Pt. The statistical analysis yielded a Pearson correlation of -0.661 with p < 0.01. Furthermore, correlation coefficients were computed between the overall PHS-Pt scores and the scores of the MCQLI-Pt ten questions separately. Using the Bonferroni approach to control for Type I error across the ten correlations, a p-value of less than 0.05/10 (= 0.005) was required for significance. The results from the correlational analyses demonstrated that eight out of the ten correlations were statistically significant and that seven of these correlations displayed values greater than or equal to -0.38. The correlations of both question 7 (community and services support; p = 0.099) and question 9 (spiritual fulfillment; p = 0.005) of the MCQLI-Pt with the overall PHS-Pt scores were not significant.

Correlation coefficients were also computed among the overall MCQLI-Pt scores and the ten PHS-Pt questions separately. The Bonferroni approach was again used as mentioned above. The results from the correlational analyses demonstrated that nine out of the ten correlations were statistically significant and that seven of these correlations displayed values greater than or equal to -0.375. The correlation between question 6 of the PHS-Pt (tiredness; p = 0.016) and the overall MCQLI-Pt score was not significant.

Considering the four groups separately, healthcare professionals displayed the highest correlation between overall PHS-Pt scores and MCQLI-Pt scores [r (28) = -0.754; p < 0.001], whereas the volunteers in the outpatient group presented the lowest correlation [r (28) = -0.462; p < 0.05]. The volunteers from the inpatient psychiatric and general hospital units also presented significant (p < 0.001) correlations between overall PHS-Pt scores and MCQLI-Pt scores (r = -0.747 and r = -0.662 respectively). The three groups of patients together also presented a significant correlation between PHS-Pt and MCQLI-Pt overall scores [r (88) = -0.656; p < 0.001].

### Bivariate linear regression

**Figure 1.**
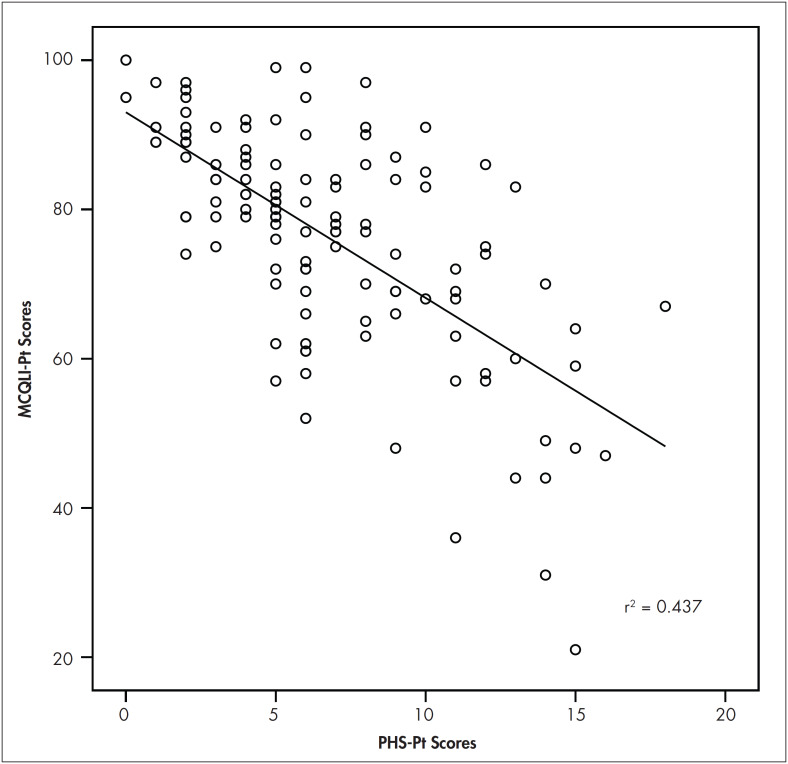
Scatter plot for the bivariate linear regression on the total scores of the Multicultural Quality of Life Index (MCQLI-Pt) and Personal Health Scale (PHS-Pt) scores.

Linear regression analysis was conducted to evaluate the prediction of the total scores of the PHS-Pt from the overall scores of the MCQLI-Pt (criterion). The scatter plot for the two variables, as shown in Figure 1, indicates that the two variables were inversely and linearly related, such that as the overall PHS-Pt scores increased, the overall MCQLI-Pt scores decreased. The regression equation for predicting the overall MCQLI-Pt scores was:

*Predicted Overall MCQLI-Pt scores = Overall PHS-Pt scores - 2.487 + 93.008*

The 95% confidence interval for the slope (-3.002 to -1.972) indicated that PHS-Pt scores were significantly related to overall MCQLI-Pt scores. As hypothesized, the subjects with high PHS-Pt scores also tended to present low MCQLI-Pt scores, which meant that participants who demonstrated poor health status (high PHS-Pt) also presented lower quality of life (low MCQLI-Pt scores). The accuracy in predicting MCQLI-Pt scores was high. As mentioned before, the correlation between PHS-Pt and MCQLI-Pt scores was -0.661. Approximately 44% (r^2^ = 0.437) of the variance in the MCQLI-Pt scores was accounted for by the linear relationship with PHS-Pt scores.

## DISCUSSION

A significant correlation between the concepts of quality of life and health status as evaluated by the Portuguese versions of the PHS and MCQLI was observed. Almost all the health-related questions presented in the PHS-Pt displayed a strong correlation with the overall concept of quality of life. In fact, the magnitude of this significant correlation explained *per se* almost half of the variance in overall MCQLI-Pt scores. These findings indicate that, within this sample, health-related issues were key factors for the overall experience of wellbeing and quality of life. Furthermore, when investigated separately, both the healthcare workers and the patients from different sources presented significant correlations between general health status and overall quality of life. This similarity indicates that the interrelation between health status and quality of life was homogenous and contiguous to different groups, regardless of the presence and/or type of ailments.

These data also indicate that community support and services as evaluated by the MCQLI-Pt was not a factor significantly related to the individual's overall perception of his own health status as evaluated by the PHS-Pt. Furthermore, spiritual fulfillment was not correlated with the overall perception of health status. In addition, tiredness or fatigue, as evaluated by the PHS-Pt, was not significantly correlated with the overall experience of quality of life. In the context of strong correlations between several physical factors, including mood, sleep and nervousness, and the overall perception of quality of life, it appears that fatigue was most probably tolerated rather than nonexistent in this sample. After all, the groups investigated in this study were composed of patients from different sources and health workers, and both types of subjects were presumably affected by some level of fatigue.

In one of the few studies displaying conceptual correspondence to ours, the health survey questionnaire SF-36 and a disease-specific (rhinitis) quality-of-life questionnaire were administered at baseline and after 12 months of intervention to 224 adults within the context of a randomized clinical trial investigating house dust mite allergy.^36^ The results revealed no significant overlapping performance between the disease-specific quality of life questionnaire and the generic health assessment tool, and the authors concluded that both types of instrument should be used simultaneously in quality of life studies.^36^ Although these results are discrepant to ours, there are meaningful methodological differences that could account for the observed differences, including the sample characteristics and research design.

In our study, we interviewed an inpatient population with levels of disease severity that were certainly greater than the levels displayed by subjects from a community sample. After all, most hospital patients experience life-threatening conditions or aggravating levels of chronic disorders. Although inpatients conceivably may be prone to value their health status as a central element of general quality of life and wellbeing, research evidence indicates that people affected by a serious health problem develop diverse reactions to changes in health status.^42^ The internal adaptation to a vulnerable health condition has been referred to as a "response shift", which is defined as a "change in the meaning of one's self-evaluation of quality of life".^43,44^ This phenomenon results from interactions between a significant health status change, personality traits and personal adaptation mechanisms.^44^

Furthermore, our study was conducted irrespective of specific diagnostic entities. Our sample was constituted of volunteers from three groups of patients admitted to general adult surgical, clinical and psychiatric units and of a control group of actively working health professionals. The above-mentioned study was a randomized clinical trial on impermeable bedding covers for combating house dust mite allergy, which contrasts with our cross-sectional study. While patients were interviewed twice in that trial, our participants were interviewed only once, with the exception of a limited fraction of the participants who were interviewed a week later as part of the respective validation protocols for the questionnaires, as described elsewhere.^37,38^

This study presents limitations that hinder subsequent interpretations regarding the possible interactions among economic, spiritual or religious factors. Although socioeconomic status and religious practice or creed affiliation could have been investigated by specific questionnaires or inquiries, we aimed to minimize the interview duration, so that both busy health workers and convalescing volunteers could easily tolerate our approach. Additionally, there was a gender disparity between the patients and health professionals in that the latter group had a smaller proportion of men. This might be related to the fact that female registered nurses and nursing assistants still outnumber their male counterparts in this region of Brazil. No gender-specific recruitment strategy was conducted in relation to any of the groups of research participants. Although such an approach could have prevented gender disproportions, it could also have artificially affected voluntary entry into the research protocol. There was a 10-year difference in mean age between the groups of patients and the health professionals. Plausibly, the subsample of professionals had a lower mean age due to the retirement cap after 25 years of professional activity, a limit that obviously does not apply to patients.

Additional studies encompassing different areas of the country as well as different cultural backgrounds might be necessary to generate a more representative picture of both regional and nationwide health standards among distinct samples of patients and the general population.

## CONCLUSION

Health-related issues were key factors for the overall experience of wellbeing and quality of life in this study. Regardless of the presence or type of disease, there was an homogenous interrelation between health status and quality of life within the groups.
